# Health Promotion in Preschool Children

**DOI:** 10.3390/children10081385

**Published:** 2023-08-14

**Authors:** Yiğit Şenol, Fatma Betül Şenol

**Affiliations:** 1 Department of Public Health, Afyonkarahisar Provincial Health Directorate, Afyonkarahisar 03030, Türkiye; 2 Department of Special Education, Faculty of Education, Afyon Kocatepe University, Afyonkarahisar 03030, Türkiye; fatmabetulsenol@gmail.com

**Keywords:** health education, health, preschool children

## Abstract

The key for individuals to live in health and well-being is the development of healthy living habits. In order for a healthy life habit to be formed, the relevant behaviors must be acquired in the preschool period. This study aimed to promote health knowledge among children attending preschool through health education. The study was designed using the qualitative case study design. The Personal Information Form and Health Hunter Children Follow Health Interview Form were used as data collection tools in the study. Health education activities were implemented in order to improve children’s knowledge about health. Activities consisted of physical activity, personal hygiene, injury prevention, sleeping habits, healthy nutrition, healthy life, and paramedic and treatment services categories. Activities were applied to children twice a week for eight weeks. As a result of the study, an improvement was observed in children’s knowledge about health. The answers given by the children before and after the Health Education Activities were collected in the categories of nutrition, safe life, illness status, well-being, hygiene, environmental health, and sports.

## 1. Introduction

Children’s cognitive, emotional, social, motor, and language development are supported and their healthy growth is ensured during the preschool age [1]. Preschool provides unique opportunities for children’s health and is important to their health and well-being throughout their life [2,3].

According to the World Health Organization (WHO), health is “a state of complete well-being, not merely the absence of disease or ill health, but also physical, mental and social well-being.” [4]. In order to lead a healthy life from the preschool age, individuals have to improve their health by increasing their control over their own health. Health promotion includes a social and individual process and aims to achieve the optimal health level of individuals from an early age [5]. Achieving optimal health is expressed as a holistic dynamic balance between physical, emotional, social, spiritual, and intellectual health [6].

In recent years, health has shifted from the biomedical model, where health and illness are polar opposites, to a holistic model of health that combines social, behavioral, and psychological aspects [7]. In the salutogenic perspective, health is seen as a state, defined as being at the opposite pole to illness but on a continuum. In this perspective, health is seen as a condition to get rid of illness or to be healthier [8]. In the multidimensional perspective, health and illness can exist simultaneously in individuals as two different dimensions. In this perspective, health and illness are not states of affairs, but the products of an ongoing process [9]. Children should be taught from an early age what needs to be done to be healthy and not to be ill. Teaching health information to children should take into account their developmental level and environmental and personal factors. Because the understanding of complex concepts such as health develops slowly in the preschool period and this development is gradually reflected in health behaviors and attitudes over time [10].

The health responsibilities of preschool children are usually under the control of their parents. Children lay the foundation for a healthy life in the future under the guidance of their parents in the preschool period. However, in the preschool period, children should especially take responsibility for hygiene and be aware of this [11]. In this direction, children should be supported at a level at which they can take their health responsibilities from the preschool period.

In Turkey, preschool children are educated in public and private kindergartens affiliated to the Ministry of National Education. This education is planned with the Preschool Education Program for 36–72-month-old children determined by the Ministry of National Education (MoNE). In the program, activities such as art, play, language, drama, literacy preparation, music, and movement are created in line with the achievements and indicators within the scope of cognitive, language, social emotional, motor development, and self-care skills [12]. In MoNE, knowledge, skills, and behaviors related to health education are acquired through activities based on achievement indicators within the scope of other development areas, especially self-care skills. However, in order for an education given in the preschool period to be permanent, the education should be systematically planned and regularly implemented. The preparation of health education programs from the preschool period for health promotion will have a positive effect on the healthy development of children.

It is necessary to prepare and design a health education program in order to protect, sustain, and develop children in the preschool period. Preschool education institutions contain unique opportunities for children’s health [2]. For this, healthy lifestyles of children should be established from the preschool period and health education should be added to preschool education [13]. Supporting children’s perceptions of health enables them to be healthy individuals in adulthood [3] and to initiate health habits from the preschool period [13]. As a result of the meta-analysis examining the health interventions in the preschool period, it was concluded that the health interventions improved the health practices of the preschool children. However, it was concluded that the interventions in the studies should be deepened, their effectiveness should be increased, their continuity and follow-up should be increased [14]. This study aimed to promote health knowledge among children attending preschool.

## 2. Materials and Methods

### 2.1. Research Design

The study was designed with a qualitative case study design. The qualitative case study investigates the factors affecting an event/situation. In the study, a single analysis unit and situation were studied. Therefore, holistic single case study design, which is one of the case study types, was used [15].

### 2.2. Study Group

The study included 23 preschool children (47.82% girls, 52.18% boys) aged 60–72 months. The children lived in the urban core of Afyonkarahisar, Turkiye, with 78.26% of them having employed mothers. All of their fathers had jobs, and the families were from middle-income backgrounds.

Criterion sampling was used to determine the participants. The basic criteria were that the children were attending preschool, were between the ages of 5 and 6, had not received health education other than the preschool education curriculum, and had Internet access and a computer/tablet at home. As the research was conducted during the COVID-19 pandemic when schools were closed and education was being delivered online, access to technology was one of the criteria considered.

### 2.3. Data Collection Tools

The Personal Information Form and Health Hunter Children Follow Health Interview Form were utilized as data collection tools.

#### 2.3.1. Personal Information Form

The form consists of questions about the demographic characteristics of children and their families.

#### 2.3.2. Health Hunter Children Follow Health

“Health Hunter Children Follow Health” is an interview form developed by Bilir Seyhan [16]. The aim of the form was to uncover the perceptions of health and health concepts by preschool children. Additionally, the aim was to examine the preschool children’s ability to explain concepts related to health using a holistic approach.

There are nine questions in the interview form. These questions are as follows: “What do you think health is?”, “What comes to your mind when you think of health?”, “Can you tell me the names of two friends who you think are healthy?”, “Why do you think these friends are healthy?”, “What do you do to be healthy?”, “What can happen in a healthy person and environment?”, “What do you see around you, healthy and unhealthy?”, “What are the things that spoil our health?”, and “What do you do when you are not healthy?”.

### 2.4. Health Education Activities

The researchers developed Health Education Activities (HEAs) to enhance the health concepts and perceptions of 60–72-month-old children with a holistic approach. The activities were produced by integrating health and education perspectives, as one researcher has expertise in preschool education, and the other in public health.

Our study team reviewed the literature before creating the HEAs. The literature categorizes health education for preschool children into eight categories: physical activity, personal hygiene, injury prevention, sleep habits, healthy eating, healthy living, health services, and treatment services [17,18,19]. Under these categories, two activities were prepared for each category.

The researchers prepared HEAs using the MoNE’s acquisition, indicators, concepts, activity types, and formats, and by integrating Turkish language, drama activities, to be suitable for online application as schools were closed during the COVID-19 pandemic. The sixteen activities were created within the scope of HEAs. Three experts evaluated and modified the activities. A pilot study was conducted prior to deploying the activities. We evaluated the online applicability, comprehensibility, and suitability of the activities in a preliminary trial involving children. Then, we finalized the HEAs by taking care of the necessary arrangements.

#### Implementation of Health Education Activities

The children received HEAs twice a week for eight weeks. HEAs were implemented with the Zoom application and small groups (consisting of 11 and 12 children) to increase interaction with the children. The groups remained unchanged until the end of the training. Each session lasted approximately 30 min. A parent accompanied the child during the training. Before the training started, technical features such as audio and video were tested. During the training, the children’s progress was monitored. Children’s opinions were frequently sought during the training to maintain their attention. Training included the use of visuals, sounds, and music. At the conclusion of each session, the children were thanked for participating in the ‘Health Hunter’ task, reminded of the upcoming activity’s schedule and bade farewell.

### 2.5. Data Collection

The data were collected online due to the current study being conducted during the COVID-19 pandemic. Moreover, schools were closed. We determined suitable days and times for the children and one of their parents. We conducted one-on-one video interviews with the children via Zoom application, during which we asked them the questions in the interview form. During the interview, the researchers informed the children that they had prepared a game for them and the children needed to answer questions to play the game. Subsequently, the questions were provided to children who wished to continue playing. As a result, the interview with the children was conducted in a playful and engaging manner, which improved the children’s attention and participation. Conversely, the children’s responses were documented on a sheet of paper. The pre- and post-interviews were conducted one week before and after the Health Education Activities, respectively, under the given conditions. Each child was interviewed for approximately 12 and 25 min for the pre- and post-interviews, respectively.

### 2.6. Data Analysis and Rigor

The data were transcribed into text as the initial step in the data analysis process. We used a conventional content analysis approach, where we created a coding scheme directly from the textual data [20]. We followed the four data analysis steps described by Green et al. [21]: data immersion, coding, creating categories, and identifying themes.

During the data immersion step, we re-listened to, transcribed, read, and re-read the interview transcripts. In this step, the researchers read and revisited the interviews and validated them through careful listening.

During the coding step, the interviews were broken down into small units, which were then integrated to form meaningful units. In this step, the interviews were systematically categorized and coded. These codes were entered into tables for later analysis. This approach ensured a more comprehensive analysis of the significant interview units.

During the category creation step, systematic and intuitive categories are formed by applying codes. Thorough analysis was performed on all codes. Related codes were merged, resulting in the formation of categories.

During the theme identification step, the categories were combined. During this stage, categories were merged, resulting in the theme of “Health”.

#### Rigor

To ensure research rigor, we carried out triangulation, expert review, participant confirmation, long-term interaction, and analysis. We maintained the data’s integrity and coder agreement. All participants were interviewed and triangulation was performed, participants’ answers to questions were confirmed after the interviews, and interaction was kept active during the interview process. Coding, categories and themes were also examined by other experts. Additionally, we analyzed the data without altering or commenting on its nature. This study used the coding “C” to refer to children in the analysis. Two different researchers examined themes and the agreement was 0.90 with Miles and Huberman’s agreement formula [22]. Since the percentage of agreement was higher than 0.70, the results obtained were considered to be reliable. The themes that did not fit were discussed with the researchers and organized.

## 3. Results

**Figure 1:**
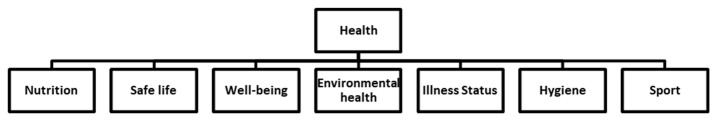
Perceptions of children about health before and after health education activities.

The perceptions of the children about health before and after the Health Education Activities were collected under the themes of nutrition, safe life, well-being, environmental health, illness status, hygiene, and sports (Figure 1).

### 3.1. Nutrition

Before the Health Education Activities, children mostly expressed health in terms of “Nutrition” theme (f = 74) (Table 1). The children made expressions such as healthy food and drink, healthy nutrition, and eating. The opinions of the children before the activities in this theme were implemented are as follows: “C1: Health is eating fruit, drinking syrup, meal.”, “C7: Oranges and carrots are healthy.”, “C13: Health is eating healthy and useful foods.”, “C20: I eat healthy to be healthy.” After the Health Education Activities, the children again mostly expressed health in terms of the “Nutrition” theme (f = 92) (Table 1). Children expressed concepts such as healthy food and drink and healthy nutrition. The opinions of the children after the activities in this theme were implemented are as follows: “C17: We should eat regularly for health, to grow up, to be hardworking.”, “C19: We should eat and drink healthy foods and have a healthy diet because it is good for our body.”, “C4: We should pay attention to eat healthy foods for healthy development.”, “C24: Being healthy means eating regularly, eating healthy foods and staying away from unhealthy junk food.”.

After the health education activities, the theme of nutrition was expressed more than before and the content of the theme was diversified and enriched. This increase and enrichment is an indicator of educational effectiveness.

### 3.2. Safe Life

Before the Health Education Activities, children expressed health in terms of the “Safe Life” theme (f = 35) (Table 1). Within the scope of the safe life theme, children expressed health as not getting sick, using medicine, and protection from germs. The opinions of the children before the activities in this theme are as follows: “Q22: Health means protection from germs and not getting sick. C5: Medicine is used for health. C21: Health means taking care not to get sick.” After the Health Education Activities, children expressed health in terms of the theme of “Safe Life” (f = 86) (Table 1). Within the scope of the safe life theme, children expressed health as not getting sick, using medicine, protection from germs, using masks, not entering crowds, protection from dangers, being safe, and not getting injured. The opinions of the children before the activities in this theme were implemented are as follows: “C8: For health, I protect myself from germs, try not to get sick, and wear a mask.”, “C11: Being healthy means not going into crowds, not getting germs, and wearing a mask. It is also to be safe.”, “Q1: Health is to stay away from dangers, not to play games where we can get injured.”, “Q16: Health is not to get sick, to sleep regularly, to recognize dangers.”.

Giving detailed information about prevention methods after the training may be an indication that children have assimilated health-promoting behaviors. This is the most important goal of education programs. Especially since the study was conducted during the COVID-19 pandemic period, the fact that they especially emphasized measures such as the use of masks for COVID-19 and staying away from crowds was found to be significant in terms of showing the effectiveness of health education to be given during pandemic periods.

### 3.3. Well-Being

Before (f = 35) and after (f = 56) the Health Education Activities, children expressed health in terms of the theme of “Well-being” (Table 1). Within the scope of the “State of Well-being” theme, while children expressed health as strength/resilience, being healthy, and being well in the pre-interview, they expressed it as feeling good/happy, being well, and not being stressed in the post-interview. The opinions of the children before the activities in this theme were implemented were as follows: “C5: Being healthy means being strong.”, “C13: Health is being healthy.”, “C19: We look good when we are healthy.”. The opinions of the children after the activities in this theme were implemented were as follows: “C2: Health means being resistant to diseases and having strong muscles. Healthy children are happy.”, “C14: Health is feeling good, well-being, that is, happiness.”, “C17: When people are not stressed, their health does not deteriorate. They feel good.” Q22: Health is everything. It is the world, happiness, joy. It is life, everything.”.

The health mentioned in the definition of health refers not only to physical health but also to a state of complete well-being. The fact that the children emphasized this after the training was evaluated as an indicator that the children reached the idea of more comprehensive well-being instead of physical health.

### 3.4. Environmental Health

Before (f = 9) and after (f = 48) Health Education Activities, children expressed health in terms of “Environmental Health” theme (Table 1). Children’s views on the theme of Environmental Health before and after the activities were expressed as not littering and clean healthy air and environment. Although there is no difference in the issues emphasized by children about environmental health, the increase in frequency and scope of their expressions was noteworthy. Sample expressions of the children about environmental health in the preliminary interviews were as follows: “C6: A healthy environment is an environment that is not polluted.”, “C15: A healthy environment has clean air.”, “C20: In a healthy environment, people do not throw rubbish on the ground.”. Their statements in the post-interviews are as follows: “C17: In a healthy environment, everywhere is clean, garbage is not thrown on the ground, there is no air pollution.”, “C8: In a healthy environment, there is silence, there is no noise and garbage.”, “C13: In a healthy environment, people are healthy, clean air means health. Everywhere is clean.”.

The awareness of the children that they need a healthy environment to be healthy was high. The fact that the children gave more importance to a healthy environment after the training shows the effect of the training.

### 3.5. Illness

Before (f = 15) and after (f = 32) the health education activities, children expressed health in terms of the theme “Illness” (Table 1). Regarding this theme, the children made statements about going to the doctor/hospital, taking medicine, and resting/not going out in the pre- and post-interviews. However, there was an increase in the expressions in the post-interview. Sample expressions of the children in the pre-interview are as follows: “C2: We go to the doctor when we get sick.”, “C9: Drinking syrup.”, “C14: Sleeping at home, not going to school.”. Sample expressions of the children in the post-interview are as follows: “C7: When we get sick, we go to the hospital to be healthy, we drink the medicines given by the doctor.”, “C13: In illness, it is necessary to lie down and rest, drink medicine.”, “C19: I do what the doctor says and rest to get better.”.

The children’s views on the perception of health in the case of illness improved after the activities. This result is important in terms of showing that children can make conscious behaviors to be healthy again in case of illness and the effectiveness of education.

### 3.6. Hygiene

Before (f = 11) and after (f = 42) the Health Education Activities, the children expressed health in terms of the theme of “Hygiene” (Table 1). Regarding this theme, children expressed hand washing, bathing, being clean, and nail cutting in the pre- and post-interviews. However, there was an increase in the expressions in the post-interview. Sample expressions of the children in the pre-interview are as follows: “C3: I wash my hands frequently to be healthy.”, “C6: I cut my nails to prevent illness.”, “C16: I always take a bath to be healthy.” Sample statements of the children in the post-interview are as follows: “C10: In order to be healthy, we should pay attention to cleanliness, that is, washing our hands, taking a bath, brushing our teeth every day.”, “C19: Health means soaping our hands until the end of the song.”, “C12: We should pay attention to hand hygiene for health because we eat everything with our hands, the germs on our hands enter our body.”.

The children pointed out that the key to being healthy is cleanliness. They include the hands, the body, and dental hygiene for cleanliness. Although these findings are important in terms of the effectiveness of education, care should be taken to provide more detailed expressions about cleanliness.

### 3.7. Sport

Before (f = 5) and after the implementation of Health Education Activities (f = 23), the children expressed health in terms of the theme of “Sport” (Table 1). While the children’s expressions related to this theme in the pre-interviews were only about doing sports/sports, in the post-interviews, they were about doing sports/sports, jogging, and exercise/gymnastics. Sample statements of the children in the pre-interview are as follows: “C18: Doing sports for health.”, “C3: Those who do sports are healthy.”. Sample statements of the children in the post-interview are as follows: “C15: Regular sports should be done for a healthy life.”, “C21: Continuous exercise helps us to be healthy.”, “C23: In order to be healthy, it is necessary to eat well, to be protected from diseases and to do sports. Jogging, going to gymnastics can also be done.”.

After the training, the children provided more information about the relationship between health and sports, which showed that the children were aware of the importance of sports for health.

## 4. Discussion

The aim of the study was to develop the health-related knowledge of children who continue preschool education. As a result of the study, children’s knowledge about health improved. The answers given by the children before and after the Health Education Activities were collected in the categories of nutrition, safe life, disease status, factors that impair health, well-being, hygiene, environmental health, sports, and living things. The children’s responses after the health education were more comprehensive.

The key for individuals to live in health and well-being is possible with the development of healthy living habits. In order for the habit to be formed, the relevant behaviors must be acquired in the preschool period. Therefore, the development of healthy living habits is possible with the acquisition of healthy behaviors in the preschool period [23].

Healthy development is primarily possible with adequate and balanced nutrition [24]. After the nutrition education was given to the preschool children, there was an increase in children’s healthy nutritional knowledge, choices [25], and healthy eating behaviors [26]. As a result of the nutrition education program called “The Snack Pack Project”, children’s knowledge of healthy nutrition increased [27]. As a result of the study conducted by Aktaç, Kızıltan, and Avcı [28] to examine the effect of family participation in the nutrition education intervention on the nutritional status of children, the positive eating behaviors of children increased. The results of studies related to nutrition education were compatible with the increase in children’s views on healthy eating and avoiding unhealthy foods obtained from this study. In line with this result, the health education provided positively affected the children’s views on healthy nutrition.

In order to lead a healthy life, children should know how to live safely from the preschool period. A safe life is to be protected from injuries and dangerous situations. The third priority of the Hyogo Framework for Action was education and information so that children can lead a safe life [29]. In order to maintain a safe life, children’s knowledge of safe living should be developed starting from the preschool period. Safe life education given to preschool children positively affected children’s safe life behaviors [30]. In the meta-analysis study conducted by Orton et al. [31], it was stated that the intervention was effective as a result of the intervention studies on safe living behaviors. The results of studies on safe life education were consistent with the increase in children’s views on safe living obtained from this study. The health education given had a positive effect on children’s views of safety.

Awareness of the factors that impair health and the state of illness in children is a situation that should be developed from infancy. Children should be given detailed education about the factors that impair their health in the preschool period [32]. In the studies, it was emphasized that the practices of the schools and the education provided contribute to the health status of children [33]. A respiratory infectious disease prevention program for preschool children was effective in changing children’s knowledge and attitudes about protection from respiratory infectious diseases [34]. Similarly, in this study, the knowledge of the health education given to children about the factors that impair health and disease states increased.

As a result of the health education activities implemented in this study, children’s views on well-being increased. Well-being for a healthy life is a situation that is managed by individuals and that is important to gain at an early age. For this, children should be educated on how to ensure well-being throughout their lives, starting from the preschool period. As a result of the “Development of Well-being in Preschool Children Program” prepared by Nithya Poornima et al. [35], there had been an improvement in the well-being of children. In the studies, quality preschool education programs including health offered to children were effective in improving children’s well-being [36]. Education given to children on health has had an impact on their well-being.

Hygiene education in preschool protects children from diseases [37] and has a high priority throughout life [38]. In the study, as a result of health education activities, children’s views on hygiene increased. In a study in which water, sanitation, and hygiene education was applied to children, children’s hygiene behaviors increased [39]. In a study in which children were educated about hand hygiene, the handwashing rates of children increased [40]. As a result of a study in which they aimed to improve their personal hygiene by reading books to children, there was an improvement in children’s personal hygiene behaviors [41]. Hygiene education given to children could be effective on children’s personal hygiene behaviors and knowledge.

Environmental health is another factor that has an impact on health. According to the WHO, environmental health is a discipline that includes these aspects of human health, including the quality of life and social well-being determined by chemical, biological, social, and psycho-social factors as well as physical environmental factors. Environmental health should be integrated into education through health education [42]. In this direction, there had been an increase in children’s views on environmental health through health education activities in the study. In a study, attention to environmental health was effective in reducing children’s diseases and disabilities [43]. Having positive behaviors and opinions about environmental health in increasing their health levels is important for children [44].

In the study, health education activities positively affected children’s views on sports. Preschool education environment has an important place in improving physical activity. In studies conducted, physical activity applied to preschool children has had a positive effect on children’s health [45]. Timmons et al. [46] and Gordon et al. [47] stated in their meta-analysis that physical activity was effective on children’s health. In this direction, offering regular physical activity opportunities to preschool children was effective in improving the healthy behaviors of the children.

## 5. Conclusions

Despite the importance given to preschool education in many countries, it is stated that all subjects such as health, nutrition, hygiene, safety, and sensitive care are not adequately supported for the healthy development of children [48]. This situation causes children to delay their knowledge of health and makes it difficult for them to make healthy behaviors a habit. In this direction, holistic health education activities applied to children have had an impact on the diversification and elaboration of children’s views on health.

## Figures and Tables

**Table 1:** Distribution of themes according to pre-interview and post-interview.

Themes	Pre-Interview		Post-Interview	
	n	%	n	%
Nutrion	74	40.2	92	24.3
Safe Life	35	19.0	86	22.7
Well-being	35	19.0	56	14.8
Enviromental health	9	4.9	48	12.6
Illness	15	8.2	32	8.4
Hygiene	11	6.0	42	11.1
Sport	5	2.7	23	6.1
Total	184	100.0	379	100.0

## Data Availability

The manuscript contains all data. The datasets used and/or analyzed in this study, however, are available upon reasonable request from a corresponding author.
